# Towards a multilevel model of major depression: genes, immuno-metabolic function, and cortico-striatal signaling

**DOI:** 10.1038/s41398-023-02466-7

**Published:** 2023-05-19

**Authors:** Elisabeth R. Paul, Lars Östman, Markus Heilig, Helen S. Mayberg, J. Paul Hamilton

**Affiliations:** 1 Center for Social and Affective Neuroscience, Department of Biomedical and Clinical Sciences, Linköping University, Linköping, Sweden; 2 Center for Medical Imaging and Visualization, Linköping University, Linköping, Sweden; 3 Department of Psychiatry, Region Östergötland, Linköping, Sweden; 4 Icahn School of Medicine at Mount Sinai, New York, NY USA; 5 Department of Biological and Medical Psychology, University of Bergen, Bergen, Norway

**Keywords:** Clinical genetics, Depression

## Abstract

Biological assay and imaging techniques have made visible a great deal of the machinery of mental illness. Over fifty years of investigation of mood disorders using these technologies has identified several biological regularities in these disorders. Here we present a narrative connecting genetic, cytokine, neurotransmitter, and neural-systems-level findings in major depressive disorder (MDD). Specifically, we connect recent genome-wide findings in MDD to metabolic and immunological disturbance in this disorder and then detail links between immunological abnormalities and dopaminergic signaling within cortico-striatal circuitry. Following this, we discuss implications of reduced dopaminergic tone for cortico-striatal signal conduction in MDD. Finally, we specify some of the flaws in the current model and propose ways forward for advancing multilevel formulations of MDD most efficiently.

Over the last half-century, in-vivo imaging and biological assay techniques have developed at a rapid pace. These techniques have made visible in mood disorders a broad variety of biological processes that were previously inaccessible. While a rough theoretical scaffolding has guided biological inquiry into mood disorders, most of this research has been exploratory given the complexity of the systems in play. More recently, and with many investigations of mood disorders conducted, several important regularities have emerged across biological levels including genetics, endocrine and cytokine signaling, and metabolomics, in addition to the neural-systems level as assessed with molecular and functional neuroimaging. In this review, we present the status of a developing multilevel biological perspective on major depressive disorder (MDD). We show that it is now possible to present an integrative thread from genetics to cytokines to neurotransmitters up to the neural-systems level in MDD. In presenting this formulation, we hope to show that cross-level integration in MDD is necessary in the conceptualization of this disorder.

Developing a biologically coherent understanding of major depression must first take into account the marked heterogeneity of MDD, given that putative depressive subtypes have shown distinct biological profiles [1]. Subtyping of MDD is both challenging and still in development [2, 3], with further progress entailing iterative refinement through interactions between the domains of behavior, biology, and nosology. The DSM-IV and DSM-5 have specifiers for melancholic and atypical subtypes of depression with the DSM-5 including a new specifier for depression “with anxious distress” [4, 5]. Importantly, severity of depression is emerging as an important dimension in the subtyping of MDD, with severe depression having a unique genetic signature [6] and clinical course, regardless of melancholic or atypical features [7]. To make this review tractable, we will focus on presenting a multilevel biological model that tends more toward atypical depression, which is distinct from melancholic and anxious depression in that increased appetite, hypersomnia, and rejection sensitivity occur in this subtype of MDD [4]. Among currently available diagnostic categories of depression, we use atypical MDD as the point of departure for our biological narrative both because atypical depression is the most heritable [8] and because it is closest to an emerging immuno-metabolic conceptualization of MDD [9]. Importantly, in developing this account, we include investigations both of atypical MDD and MDD more broadly construed when variables examined have high relevance to atypical depression (e.g., metabolomic and immunological studies).

## Genetics of depression

In spite of evidence from twin-concordance studies for moderate (33–42%) heritability of MDD [10], early genome-wide association studies (GWAS) of depression did not identify genome-wide-significant single nucleotide polymorphisms (SNPs) associated with this disorder [11]. As the size of case and control samples grew, the power of GWAS studies improved and significant variants were detected [12–15]. Correspondence between detected SNPs across these studies, however, was low with only one common depression-associated SNP identified across any pair of studies [12–15]. A recent meta-analysis of GWAS studies of depression, however, identified stronger and more consistent findings, with 102 genome-wide significant variants detected, 56 of which were found to be genome-wide significant in an independent replication cohort [16].

This most recent MDD GWAS identified a strong genetic link between MDD and metabolic dysfunction. This link is most readily seen from the results of genetic correlation analysis for estimating the degree of genetic correspondence between depression and a variety of traits. Among 31 traits found to have a significant, positive genetic correlation with MDD, nine were from the domain of metabolic disturbance (obesity class 3, body fat, triglycerides, coronary artery disease, waist-to-hip ratio, overweight, waist circumference, obesity class 1, and body mass index).

## Metabolic disturbance and depression

The link between the genetics of MDD and metabolic disturbance is not surprising given consistent evidence connecting MDD and metabolic syndrome, a syndrome characterized by abdominal obesity, increased fasting-glucose levels, high blood pressure, high triglyceride levels, and decreased high-density lipoprotein cholesterol [17]. For example, a meta-analysis of 29 epidemiological cross-sectional and prospective longitudinal cohort studies showed a bidirectional relation between MDD and metabolic syndrome such that depression predicts metabolic syndrome and vice versa [18]. While the potentially many pathophysiological mechanisms connecting metabolic syndrome and depression have not been extensively investigated, one promising candidate is insulin resistance. While the brain was long considered an insulin-independent organ, more recent work has shown that insulin receptors are distributed abundantly in the brain [19] and that pancreas-derived insulin binds to epithelial cells on the blood-brain barrier and can then enter the brain via receptor-mediated transcytosis [20]. Importantly, brain cells expressing insulin receptors also show insulin insensitivity [21]. The implications for neural-level insulin resistance and MDD are clear considering findings that obese humans show neural unresponsiveness to exogenous insulin [22] and fail to show mood elevating effects [23] observed in normal-weight humans resulting from a course of intranasal insulin administration [24].

In the context of metabolic disturbance in MDD, it is important to consider a potential role for the gut microbiome in the pathophysiology of depression. Several putative links between MDD and gut-microbe composition are under consideration [25]. One intriguing possibility extends from microbial degradation of dietary fibers in the gut, a function which human intestinal enzymes lack. Some of the byproducts of this degradation, namely short chain fatty acids (SCFAs), are believed to be bioactive, with effects on metabolism [26]. A double-blind study assessing the metabolic and behavioral effects of dietary fiber supplementation found a lipid-lowering effect, weight loss, and increased satiety, which was associated with increases in fecal SCFAs [27]. This is a promising finding considering the increasing attention SCFAs have garnered recently as a possible biomarker, or pathophysiological substrate, of MDD [28].

Importantly, in the context of MDD, metabolic disturbances and inflammation are consistently linked. A large cross-sectional study examined waking cortisol response, inflammatory cytokine levels, and metabolic syndrome in melancholic MDD and atypical MDD. This investigation showed that metabolic syndrome and increased inflammation, but not heightened cortisol response, co-occur in atypical MDD whereas exaggerated waking cortisol response occurs in melancholic MDD in the absence of elevated inflammation and metabolic syndrome [1]. This association between atypical MDD, inflammation, and metabolic syndrome has been replicated in a large, twin-registry cohort study of metabolic syndrome [29]. Thus, extant data reveal strong links between the genetics of depression, metabolic disturbance, and inflammation.

## Inflammation, dopamine, and depression

Inflammation is a protective response of the body to pathogens or injury. Researchers have noted the resemblance between depressive symptoms and the sickness-syndrome induced by inflammation, which is characterized by increased sleep, inactivity, and social withdrawal [30]. This, in addition to the finding that immune-activating therapies—such as interferon-alpha treatment for hepatitis C [31] and autoimmune disorders [32]—lead to elevated levels of depressive symptoms has motivated investigations of the role of inflammation in MDD. Indeed, a significant body of evidence for immune system involvement in MDD has amassed over the past four decades. These data indicate a general dysregulation of the inflammatory response in MDD with abnormalities in both pro- and anti-inflammatory chemical messengers [33–35]. While it is uncommon to treat MDD with anti-inflammatory drugs alone, augmentation of standard anti-depressants with anti-inflammatories is effective in comparison to placebo augmentation [36]. Moreover, it has been proposed that the rapid-acting antidepressant effects of the psychoactive drug ketamine could be anti-inflammatory in nature given the effects of this drug on both peripheral and central inflammation [37].

Inflammation in both the peripheral and central nervous system can lead to the activation of the brain’s microglia and dendritic cells, prompting an immune response that alters neural functioning [38]. Administration of the pro-inflammatory cytokine interferon-alpha to rats, for example, decreases central levels of dopamine and tetrahydrobiopterin [39], where tetrahydrobiopterin is an enzyme co-factor for tyrosine hydroxylase, the rate-limiting enzyme in dopamine synthesis. Additionally, activation of mitogen-activated protein kinase pathways by inflammatory challenge upregulates dopamine-transporter activity [40], reducing post-synaptic availability of dopamine. Further, results from mouse models show that inflammation-activated microglia produce prostaglandins which modulate striatal-neuron activation resulting in negative affect [41], potentially by inhibition of dopaminergic cells [42]. Similar effects have been observed in humans where increased anhedonia and depressive symptoms in response to inflammatory stimuli have been reliably related to decreased dopamine release and striatal activation [43]. Importantly, dopamine is not the only neurotransmitter implicated in the inflammation-induced signaling cascade. Inflammation has also been linked to reduced availability of serotonin [44] in addition to neuro-structural alterations [45] through the kynurenine pathway.

## Kynurenine pathway and depression

**Fig. 1:**
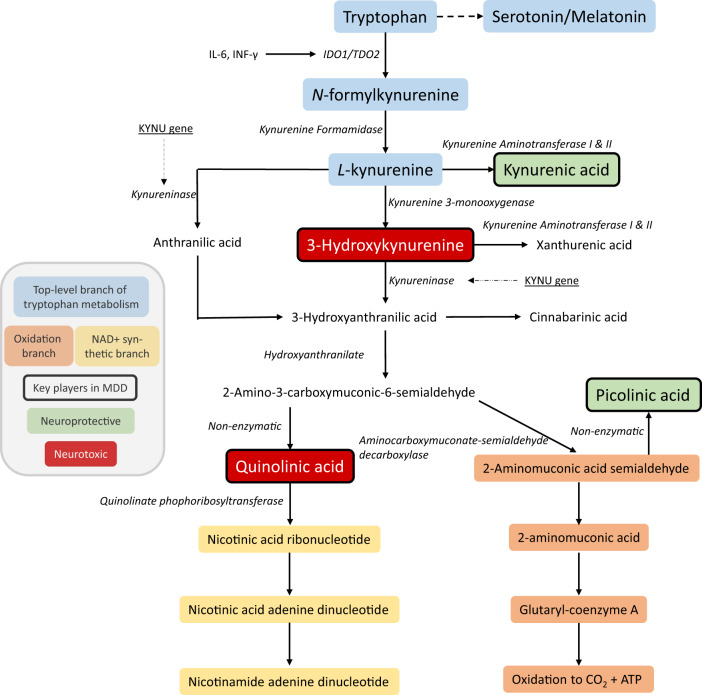
Schematic overview of the kynurenine pathway and its significance in major depression. ATP adenosine triphosphate, IDO1 indoleamine 2,3-dioxygenase 1, IL interleukin, INF interferon, MDD major depressive disorder; NAD+ nicotinamide adenine dinucleotide, TDO2 tryptophan-2,3-dioxygenase 2. Blue fields belong to the top-level branch of the tryptophan metabolism pathway, orange fields are in the oxidative branch and fields within the NAD+ synthetic branch are shown in yellow. Metabolites shown in green are considered to have neuroprotective properties while metabolites in red are considered neurotoxic. Metabolites outlined in bold are key players along the kynurenine pathway in major depression.

Of many genes previously predicted to associate with MDD, one of the few found to do so is the KYNU gene [16] which codes for kynureninase, an enzyme critically involved in the kynurenine pathway. See Fig. 1 for a schematic overview of this pathway. Along this pathway, tryptophan is converted to kynurenine (KYN) by the rate-limiting enzymes indoleamine 2,3-dioxygenase 1 (IDO1) and tryptophan dioxygenase 2 (TDO2). Pro-inflammatory cytokines, such as interferon-gamma, activate IDO1 and TDO2 [46], reducing the bioavailability of tryptophan to convert to serotonin. KYN can then be further metabolized by kynurenine aminotransferases I-IV, which catalyze the synthesis of kynurenic acid (KYNA) in astrocytes [47]. Alternatively, KYN can be metabolized by kynurenine 3 monooxygenase (KMO) to 3-hydroxykynurenine (3-HK) in microglia [46]. Here in the kynurenine pathway, the KYNU gene comes into play by coding for kynureninase, which catalyzes KYN and 3-HK to anthranilic and 3-hydroxyantrhanilic acids, respectively. In subsequent kynurenine pathway steps, these acids can either convert in microglia [48] to quinolinic acid (QUIN)—which can then become nicotinamide—or to picolinic acid (PIC). Importantly, the functional implications of having the KYNU-gene risk allele have not been well characterized in the brain or elsewhere. One recent study assessed KYNU expression in the anterior cingulate in postmortem brain tissue of 24 depressed patients and 12 never-disordered controls, finding no differences in KYNU expression between the groups [49].

Several kynurenine pathway metabolites have neuroactive effects, some of which are neurotoxic and others which are neuroprotective or neurotrophic. 3-HK is considered neurotoxic by virtue of being a generator of free radicals, whereas QUIN is excitotoxic through agonism at N-methyl-D-aspartate (NMDA) receptors [50]. Among the neuroprotective kynurenine pathway metabolites are KYNA, which, like the fast-acting antidepressant ketamine, protects neurons through competitive antagonism at NMDA receptors [51, 52], and PIC, which blocks the neurotoxic effects of QUIN [53]. Increased neurotoxic [54] and decreased neuroprotective [55] kynurenine pathway metabolite levels, and decreased neuroprotective to neurotoxic metabolite ratios [56] have been observed in plasma in MDD. We recently extended these findings in the periphery by assessing kynurenine pathway metabolite levels in the cerebrospinal fluid (CSF) of MDD patients and healthy controls [57]. We found in CSF that neuroprotective PIC and PIC/QUIN ratios were reduced in MDD as well as inversely correlated with body mass index (BMI), which was significantly elevated in MDD. Importantly, weight gain is a cardinal symptom of atypical depression and has been considered important in conceptualizing atypical MDD in terms of metabolic and inflammatory disturbance [9]. This formulation is further supported by the finding that longitudinal changes in kynurenine pathway metabolite levels and metabolic disturbances (diabetes, hypertension, and obesity) co-occur [58].

A shift from neuroprotective to neurotoxic activity in the kynurenine pathway could affect brain-structure volume in MDD. Brain areas that are reliably found to be altered in MDD, such as the hippocampus, amygdala, and striatum, are especially vulnerable to the effects of neurotoxic kynurenine pathway metabolites. Volumetric reductions in the hippocampus and amygdala, regions with a high expression of the GluN2B subunit on which QUIN acts [51], have been associated with a reduced KYNA/QUIN ratio in MDD [45]. Moreover, QUIN injection into the striatum leads to excitotoxic lesions in rodents, mostly due to loss of medium spiny neurons that predominate in this dopaminergically innervated region [59].

## Dopamine and depression

As we detail above, inflammation impacts dopaminergic function. The first speculations about the involvement of dopaminergic abnormalities in MDD conceptualized these abnormalities in terms of their relation to cognitive and behavioral stereotypies in depression, such as repetitive, unwanted focus on negative events and aspects of the self [60]. This later gave way to hypotheses of a dopaminergic deficit in MDD accounting for reduced psychomotor speed—which is especially evident in atypical depression—in addition to anhedonia and impaired concentration [61]. Enthusiasm for a dopamine-based hypothesis of depression subsequently waned, however, potentially as a response to inconsistent results from both postmortem and neuroimaging findings [61]. Nonetheless, recent data provide some cause for renewed interest in a role for dopamine in MDD.

As we have reported elsewhere [62], the striatal-dopamine neuroimaging literature in MDD is inconsistent, with the bulk of studies reporting no differences between depressed and healthy samples with respect to the binding potential of dopamine in the striatum. To bolster signal quality, most investigations conducted to date of the dopamine type-2 receptor (D_2_)—most abundant in the striatum relative to other brain regions—have averaged across anatomically defined striatal regions-of-interest. Based on functional connectivity maps of the striatum, however, this region appears to be organized at a much finer spatial scale than its anatomical boundaries suggest [63]. If we therefore limit our consideration of the literature to the five studies that conducted voxel-wise, as opposed to coarser anatomical-region-of-interest comparisons of the binding potential of striatal D_2_ receptors, a more consistent pattern of findings emerges. Three of these studies report increased binding potential in MDD [62, 64, 65], whereas one study reports reduced binding potential [66], with one study finding no difference between depressed and healthy samples [67]. It is important to consider that increased binding potential of striatal D_2_ receptors could hypothetically result from reduced competition of radioligand with endogenous dopamine, or increased striatal D_2_ receptor density, or a combination of these factors. Striatal D_2_ receptor imaging can detect altered competition with endogenous ligand. For example, injection of amphetamine, which increases extra-synaptic striatal-dopamine levels via effects on monoamine transporters, results in reduced striatal D_2_ receptor binding [68]. Conversely, in Parkinson’s disease, where dopaminergic input to the striatum from the substantia nigra is reduced, increased radiotracer binding to striatal D_2_ receptors is reliably observed [69]. Amphetamine challenge D_2_ receptor binding studies using depressed samples—with the capability of disambiguating endogenous-competition versus receptor-density accounts in MDD—are lacking, with just one pilot study reporting equivocal effects [70].

While any assertions about the coherence in the dopamine neuroimaging literature in MDD might be preliminary, more consistent findings have been reported from a recent meta-analysis of studies assaying CSF for the dopamine metabolite homovanillic acid (HVA). Unlike serotonin and norepinephrine metabolites in CSF that do not consistently differ in depressed relative to control samples, HVA levels are reliably reduced, with 19 of 23 studies reporting lower HVA in the CSF in depression [71]. As CSF measures of neurotransmitter activity are necessarily coarse, this reliable finding raises additional questions about the dopaminergic regions implicated. Nonetheless, in the domain of pharmacotherapy, a recent meta-analysis provided support for the efficacy of partial dopamine agonists—a similarly broad tool—in the treatment of MDD in treatment-refractory patients [72]. Finally, single nucleotide polymorphisms identified in the most recent, large-scale GWAS of MDD showed a highly reliable gene-based hit (*p* < 10E−13) within the DRD2 gene, which codes for the D_2_ receptor. Interestingly, no significant gene-based hits were identified for genes involved in the synthesis, post-synaptic transport, or receptor expression for serotonin. Similarly, drug-gene interaction analyses identified several hits for dopaminergic drugs and only one for a drug with direct serotonergic properties—the non-selective serotonin reuptake inhibitor vilazodone [16].

## The cortico-striatal-pallido-thalamic circuit

The cortico-striatal-pallido-thalamic circuit (henceforth referred to as the cortico-striatal circuit) represents a macro-architectural facet of neural organization. This re-entrant circuit is a major pathway through which cortical and brain-stem functions are united. The first conceptualizations of the cortico-striatal circuit proposed that it had an aggregating function, funneling a broad set of inputs from all of cerebral cortex toward a behavioral output ultimately implemented in motor cortex [73]. Data collected subsequently, however, suggested that this circuit is divided into segregated, “closed-loop” sub-circuits, with a motor circuit from sensorimotor cortex to the putamen and an association loop coming from multimodal association regions and passing through the caudate [74]. This functional differentiation was later broadened to include at least five segregated cortico-striatal circuit subdivisions linked to motor, oculomotor, dorsolateral prefrontal (DLPFC), lateral orbitofrontal, and anterior cingulate cortices (ACC) [75].

More recent work has challenged the assertion that the sub-circuits of the cortico-striatal circuit architecture are segregated. This work has been fueled by the claim that communication among the functionally specialized circuits is necessary for implementing complex, goal-directed behaviors. Consistent with this assertion, it was observed that in the striatum there are subregions of convergence between terminals from anterior cingulate, dorsolateral prefrontal, and motor regions [76]. Further, it was found that direct dopaminergic projections from the substantia nigra to cortical regions are diffuse, with individual cells in the substantia nigra sending collateral axons to parts of cortex that were putatively part of distinct cortico-striatal sub-circuits [77].

**Fig. 2:**
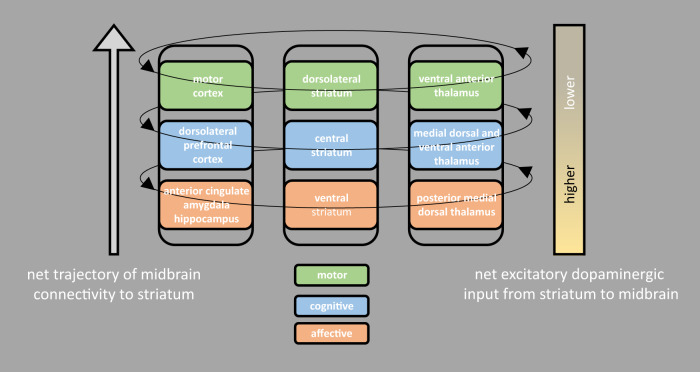
The cortico-striatal circuit. Primary components of the dopaminergically mediated, ascending spiral, cortico-striatal-thalamic circuit.

The cortico-striatal circuit has a “logic-board” configuration that determines whether signal conduction through this circuit is facilitated or inhibited. See Fig. 2 for a schematic. The so-called direct pathway is facilitatory such that cortical stimulation of the striatum blocks (via GABA-ergic striato-pallidal connections) tonic inhibition of the thalamus by the internal segment of the pallidum and substantia nigra pars reticulata, thereby enabling thalamo-cortical signal transfer. The inhibitory, indirect pathway is more complex and includes two additional terminals. Via this pathway, the cortically excited striatum sends inhibitory, GABA-ergic projections to the external pallidum which then dampens tonic pallidal inhibition of the subthalamic nucleus. The activated subthalamic nucleus then sends excitatory input to the internal pallidum/substantia nigra pars reticulata thereby facilitating its tonic inhibition of the thalamus [78].

The most important development in recent years with respect to our knowledge of the cortico-striatal architecture is that its subdivisions are not just a connected set of re-entrant, go/no-go circuits, but rather are connected in an orderly way that constitutes a “motivation-to-movement interface.” [79] The primary mechanism of this aspect of the cortico-striatal architecture is a change in bidirectional dopaminergic innervation between striatal and midbrain structures that runs along a ventral-to-dorsal gradient. Ventrally, striatum-to-midbrain dopamine innervation is maximized and midbrain-to-striatum dopaminergic connections are minimized. Proceeding dorsally, this asymmetry gradually reverses such that dopaminergic connectivity from the striatum to the midbrain diminishes and midbrain-to-striatum dopamine innervation is increased.

First, the ventral striatum—which is strongly connected with affective and reward-related regions such as the amygdala, hippocampus, orbitofrontal cortex, and anterior cingulate cortex—receives limited, and highly localized dopaminergic input from neurons in the ventral tegmental area extending to the substantia nigra pars compacta. Dopaminergic input from the ventral striatum back to midbrain regions, however, is much more topographically diffuse and includes parts of the midbrain that send dopaminergic projections to the central striatum, which receives glutamatergic projections from cortical regions implicated in higher cognitive functions. The central striatum, in turn, sends dopaminergic projections back to parts of the midbrain with dopaminergic connectivity to the dorsolateral aspect of the striatum. Finally, the dorsolateral striatum receives glutamatergic connections with motor regions and projects only limited dopaminergic connections to the midbrain. By virtue of this pattern of dopaminergic influence along an ascending spiral starting with the ventral striatum and ending with motor cortex, affective, cognitive, and motor processing are integrated [79]. See Fig. 2.

The interactions between the core cortico-striatal architecture and striatum-midbrain-striatum connections described here are not well characterized. There is evidence, however, that the dopaminergic projections from the substantia nigra pars compacta to the striatum reinforce cortical activation of cortico-striatal sub-circuits via contrasting actions on direct and indirect cortico-striatal pathways [78]. These nigrostriatal inputs appear to have a net excitatory effect on striatal neurons providing GABA-ergic inhibition of the external pallidum (facilitatory, direct pathway) and a net inhibitory effect on striatal neurons with GABA-ergic projections to the internal pallidum/substantia nigra pars reticulata (inhibitory, indirect pathway) [80–83]. Relatedly, the conventional account of the D_2_ receptor is that this G-protein coupled receptor (GPCR) is relatively fast acting and inhibitory where G_αi_ subunits bind to and block adenylyl cyclases, preventing production of cyclic AMP [84]. Nonetheless, recently there has been growing acknowledgement that D_2_ receptors can signal through beta-arrestin-2-dependent pathways and that this mode of action is complementary to canonical GPCR signaling, acting over the longer term to facilitate activity in D_2_ receptor-expressing neurons [85]. Consistent with this account, mutant D_2_ receptors that recruit arrestin but lack G-protein activity restore locomotor activity in D_2_ receptor knockout mice when expressed virally in the ventral striatum [86].

## Depression, dopamine, and the cortico-striatal circuit

In a meta-analysis of task-based functional neuroimaging studies of depression, we found reliably increased activation in MDD of the amygdala and ACC and reduced response of DLPFC to negative affective challenge. Moreover, we found reduced striatal response in MDD to both negative and positive affective provocation. We interpreted these findings in the context of the dopaminergically mediated, ascending spiral cortico-striatal circuit model detailed above. Specifically, we proposed that in MDD amygdalar and ACC inputs to the “bottom,” affective subdivision of the cortico-striatal spiral were exaggerated (potentially by increased tonic input to these regions by the pulvinar nucleus of the thalamus) and that signal to higher cortical regions was dampened due to reductions in striatal dopaminergic activity facilitating signal conduction up the cortico-striatal spiral [87]. Embedded in this interpretation was the novel, testable hypothesis that striatal regions showing reductions in dopaminergic input in MDD should also show reduced functional connectivity with their cortical targets and that this should scale with the degree of the reduction in dopaminergic tone. We tested this hypothesis by concurrently imaging D_2_ receptor binding potential using positron emission tomography (PET) and neural activity using resting functional magnetic resonance imaging in depressed and healthy comparison samples. Consistent with our hypotheses, we observed increased D_2_ receptor binding potential in depressed participants in regions of the ventral and dorsal striatum. Moreover, we saw that as binding potential increased in ventral striatum and dorsal striatum in MDD, connectivity with the respective default-mode and posterior salience network targets of these striatal regions decreased [62]. This finding provided confirmation of the ascending spiral, cortico-striatal model in that dopaminergic input to the ventral striatum predicted connectivity only with its default-mode network target (hypothesized to subserve cognitive functioning) whereas dopaminergic input to dorsal striatum predicted connectivity primarily with motoric regions of the posterior salience network. From an affective standpoint, we propose that these data indicate that in MDD the emotional aspects of negative stimuli are experienced more intensely and that layers of mitigating, contextual information provided by higher cortical regions [88] are diminished due to reduced signal propagation to these regions up the cortico-striatal spiral. Imagine, for example, seeing an image of a snake on a television and your brain over-accentuating the snake and under-representing the televised medium.

In the continued development of cortico-striatal-thalamic models in MDD it will be important to integrate habenular function. The lateral habenula exerts an inhibitory influence on dopaminergic neurons of the ventral tegmental area and substania nigra [89], which are considered important for learning by negative reinforcement [90]. Consistent with this functional role of the habenula, aberrant habenula volume [91] and functioning [92, 93] have been observed in MDD.

## Summary, limitations, and future directions

**Fig. 3:**
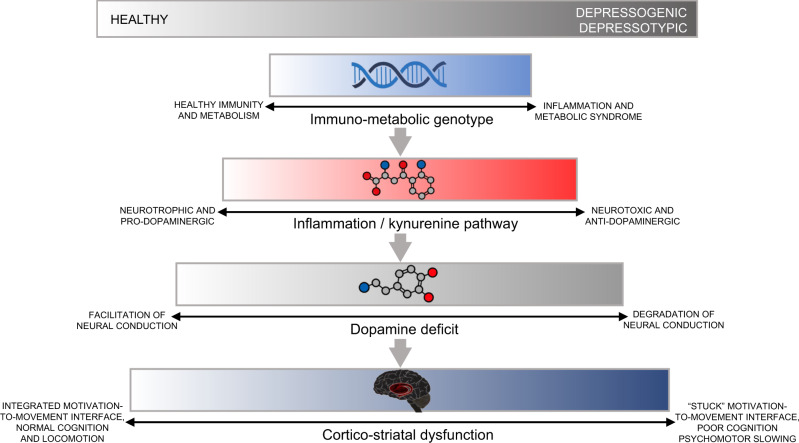
Summary schematic. Our multilevel model of major depression from genetic to cytokine signaling to neurotransmitter to neural-system levels.

Here we present a narrative connecting abnormalities from genetics to metabolic and cytokine and neurotransmitter signaling to the neural-systems level in major depression. See Fig. 3 for a summary schematic of our synthesis. The connections between these levels derive from large-scale and/or replicated investigations as well as from theoretical architectures. While the potential for such cross-level integration is encouraging, the account we present is far from comprehensive and final. For example, the connections we detail between the polygenetic signature of MDD and metabolic and immunological dysfunction extend from genetic correlations between these domains. We lack a stepwise, functional-genomics description of this link detailing the genetic-proteomic pathways involved. Moreover, much of the formulation we present connecting immunological abnormalities to dopaminergic signaling is based on pre-clinical models and awaits confirmation in humans. Further, the literature linking dopaminergic abnormalities in MDD with neural-system-level dysfunction is scant and awaits replication. Finally, while we have been careful to avoid unwarranted assertions of causality here, a hierarchical, multilevel account such as this one cannot help but contain some implied causality along the gene-to-whole-brain trajectory.

The account we present suggests at least as many testable hypotheses as it addresses in the development of a multilevel account of major depression. As we state above, formulations relating immunological alterations and dopamine signaling in MDD require more and better testing. In this context, multimodal radioligand imaging for relating immunological and neurotransmitter levels—tandem translocator protein and raclopride PET imaging, for example—in a case-control design could be useful. Further, our assertions that inflammation causes changes in dopaminergic signaling in the brain are derived only from animal-model studies. To test this causal hypothesis in humans it would be feasible to conduct an experimental medicine study assessing dopaminergic neurotransmission in individuals during inflammation challenge (e.g., with lipopolysaccharide [94]) versus placebo. Finally, while the immuno-metabolic characterization of depression has been useful, immune and metabolic factors are not completely synonymous [9] and causal investigations of the latter are largely absent. Another potentially valuable line of investigation, therefore, could focus on the development of metabolic challenge paradigms with emphasis on their neural-level effects.
